# Supplementary material for: Lymphocyte infiltration and thyrocyte destruction are driven by stromal and immune cell components in Hashimoto’s thyroiditis
Source: Nat Commun. 2022 Feb 9;13:775. doi: 10.1038/s41467-022-28120-2 (PMC8828859; doi:10.1038/s41467-022-28120-2)
Supplement: Supplementary file 1 — Supplementary Information [file 41467_2022_28120_MOESM1_ESM.pdf]

Lymphocyte Infiltration and Thyrocyte Destruction is Driven by Stromal and Immune Cell Components in Hashimoto's Thyroiditis

Supplementary information

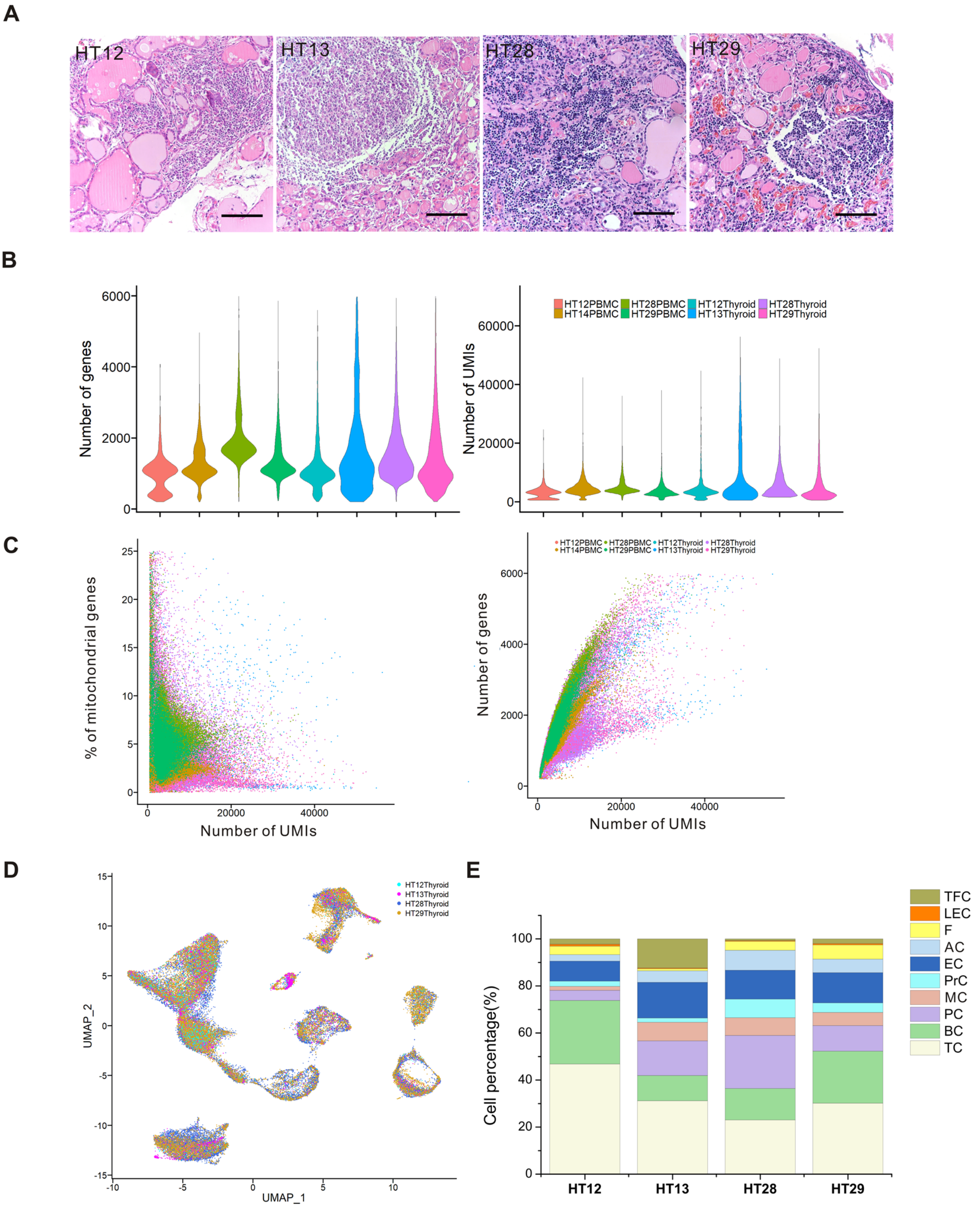

Supplementary Figure 1. Representative thyroid tissue histopathology of HT patients and cell distribution and composition of single cells from the thyroid.

(A) HE staining of the thyroid tissue from each patient showing characteristic immune cell infiltration and thyrocyte destruction in HT. Scale bar represents 100 μm. (**B**) Numbers of genes, number of UMIs, percentage of mitochondria UMIs, and UMIs per gene detected in each sample after quality control, cells with gene number>200 and <6000, and mitochondria gene percentage <25% were used in the current study. (**C**) Dot plot showing the relationship between percentages of mitochondria genes and UMIs (left) and between gene counts and UMIs (right) detected in each

sample after quality control. (**D**) UMAP of total cells of the thyroid, colored by patient ID. Cells from different patients distributed homogeneously in all the clusters. (**E**) Fractions of total cells present in each cluster for each patient were similar. TC: T cells, BC: B cells, PrC: proliferative cells, PC: plasma cells, MC: myeloid cells, TFC: thyroid follicular cells, EC: endothelial cells, LEC: lymphocytic vessels endothelial cells, F: fibroblasts, AC: ACTA2$^+$ cells, DC: dendritic cells, Mac: macrophages. NaB: naïve B cells.

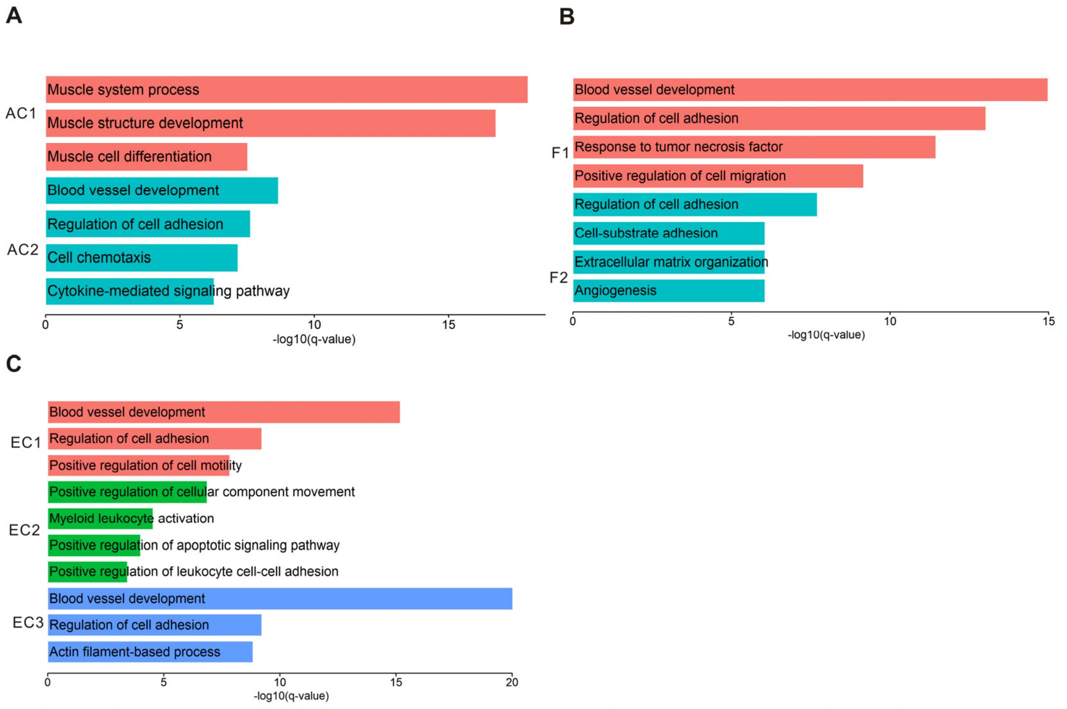

Supplementary Figure 2. GO enrichment analysis of stromal cell subgroups of thyroid tissue revealed distinct functions.

(**A-C**) Gene enrichment analysis of DEGs for subgroups of $ACTA2^+$ cells (A), fibroblasts (B), and endothelial cells (C). The statistical significance was tested by two-sided hypergeometric test and adjusted by Benjamini-Hochberg correction. F: fibroblasts, AC: $ACTA2^+$ cells, EC: endothelial cells.

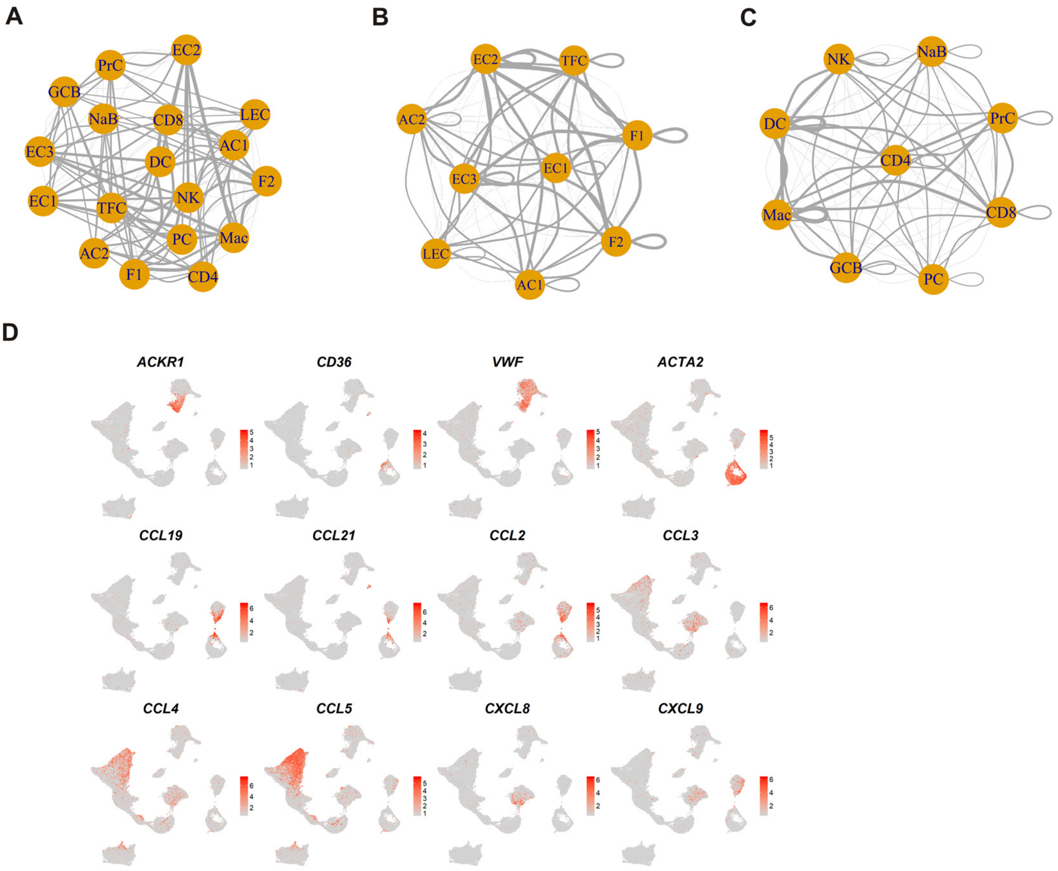

Supplementary Figure 3. Cell interaction plots between cell subgroups of the thyroid and expression of some marker genes.

(**A-C**) cell–cell communication between immune and stromal cell subgroups (A), between stromal cell subgroups (B), and between immune cell subgroups (C), visualized using the iGraph package in R. *P* values were calculated by one-sided permutation test with 10,000 permutations. Only significant interactions with *P* value <0.01 and log2 (mean) >0 were shown. Frequent interactions were found between immune and stromal cell subgroups. (**D**) Gene expression of staining markers and specific chemokines in cells of the thyroid overlaid on the UMAP visualization. TC: T cells, BC: B cells, PrC: proliferative cells, PC: plasma cells, MC: myeloid cells, TFC: thyroid follicular cells, EC: endothelial cells, LEC: lymphocytic vessels endothelial cells, F: fibroblasts, AC: ACTA2^{+} cells, DC: dendritic cells, Mac: macrophages. NaB: naïve B cells, GCB: Germinal Center B cells.

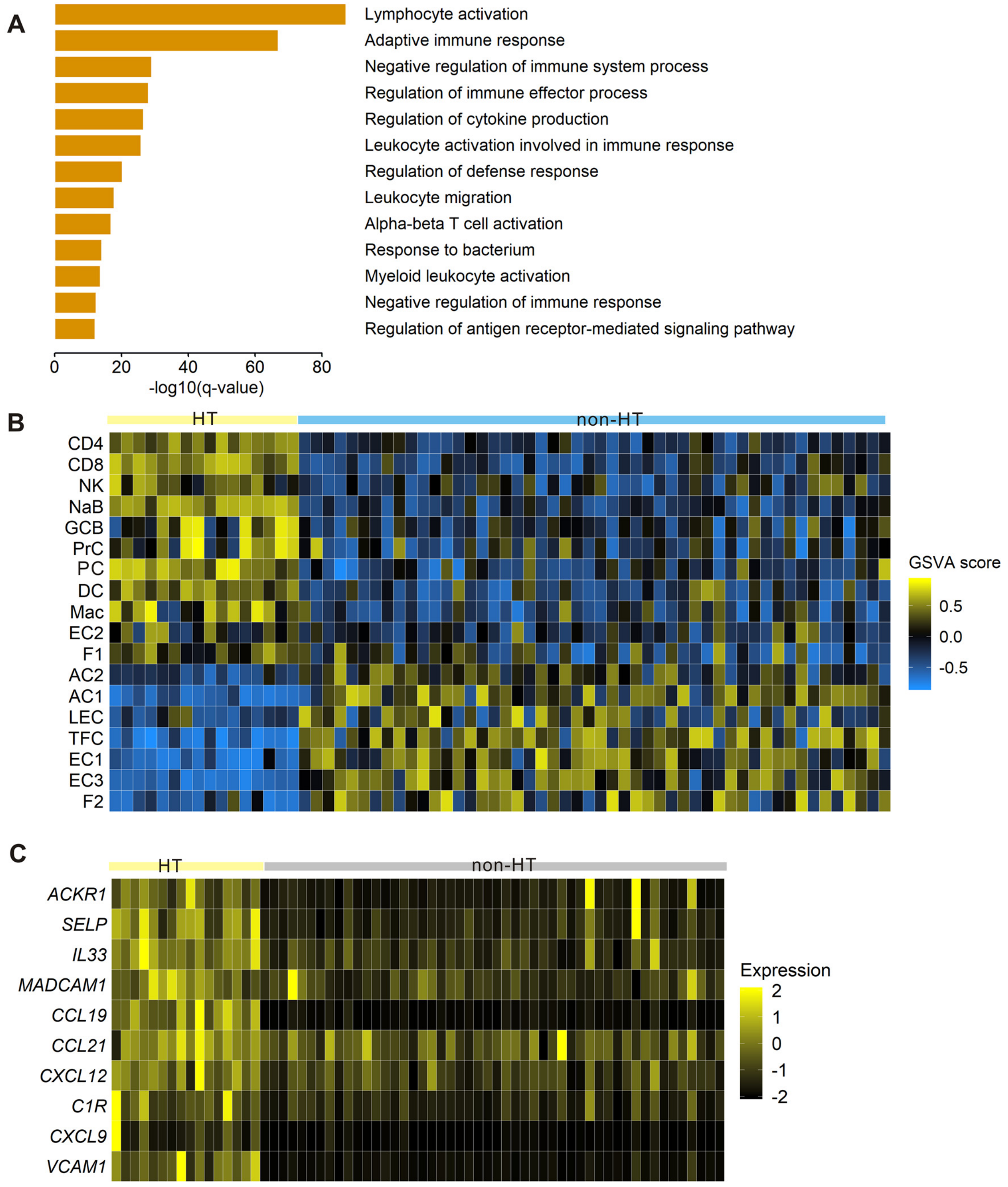

Supplementary Figure 4. Bulk RNA-seq verified the increase of CCL21-expressing stromal cells and ACKR1+ endothelial cells in HT patients.

(**A**) Enriched GO processes of differentially expressed genes in HT patients compared with non-HT patients identified by Bulk-RNAseq of the thyroid tissue. The statistical significance was tested by two-sided hypergeometric test and adjusted by Benjamini-Hochberg correction. (**B**) Heatmap of GSEA scores based on gene sets consist of marker genes of each cell subgroup as shown in figure 1E in 16 HT patients and 50 non-HT patients; each column represents one individual. (**C**) Expression levels of selected genes involved in tertiary lymphoid neogenesis in 16 HT patients and 50 non-HT patients. TC: T cells, BC: B cells, PrC: proliferative cells, PC: plasma cells, MC: myeloid cells, TFC: thyroid follicular cells, EC: endothelial cells, LEC: lymphocytic

vessels endothelial cells, F: fibroblasts, AC: ACTA2^{+} cells, DC: dendritic cells, Mac: macrophages. NaB: naïve B cells, GCB: Germinal Center B cells.

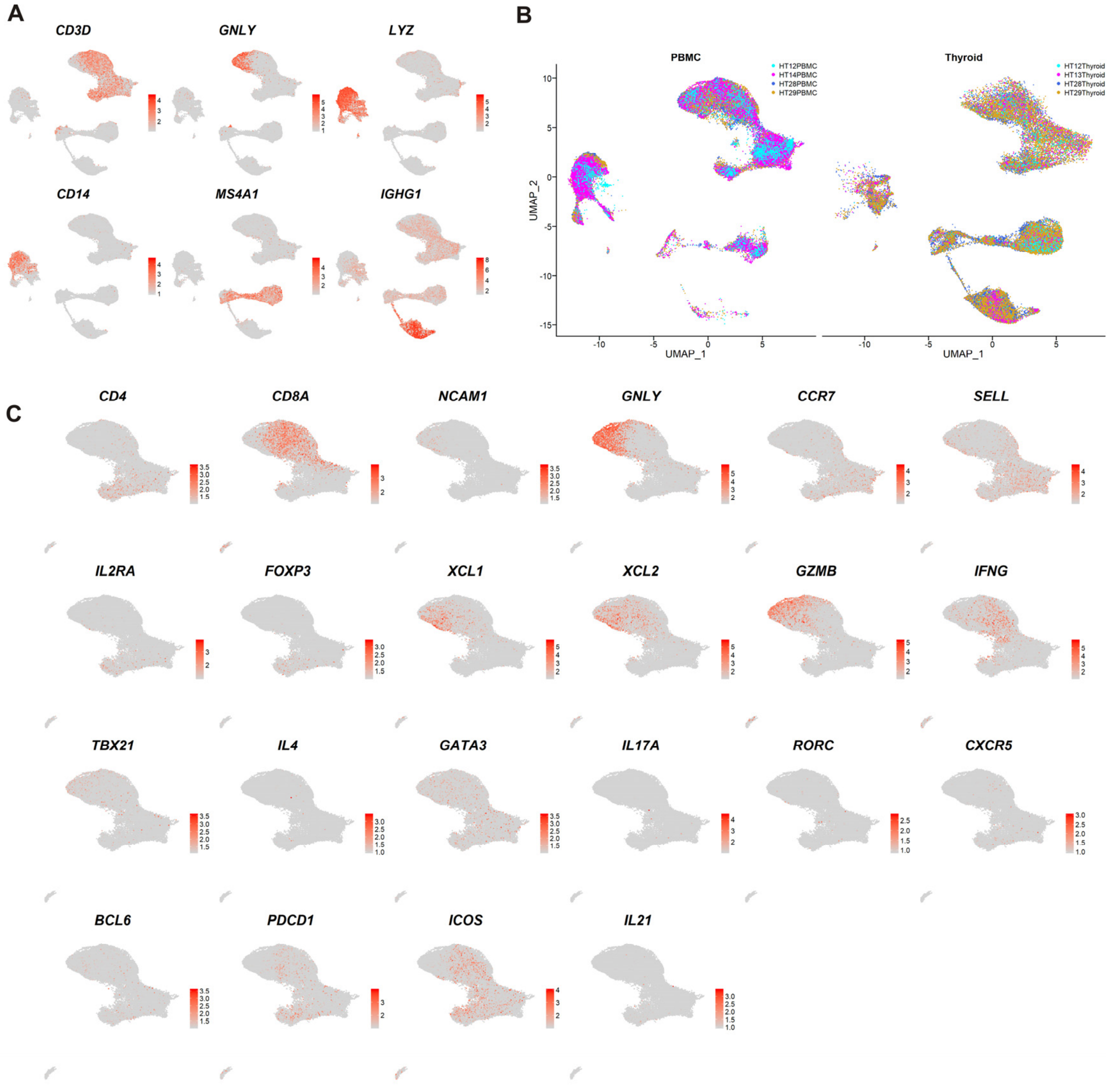

Supplementary Figure 5. Cell distribution of merged immune cells from each patient and marker gene plot of immune cell subsets.

(**A**) Gene expression of marker genes for the three major clusters of merged immune cells. (**B**) UMAP of the merged immune cells from PBMCs (left) and thyroid (right), colored by patient ID. Cells from different patients distributed homogeneously in all the clusters. (**C**) UMAP plot showing the expressions of known marker genes of T cell subtype.

Supplementary Tables

Supplementary Table 1. Sample characteristics of five HT patients for single-cell RNA sequencing

**Patient ID**	**Sex**	**Age**	**Diagnosis**	**TGAb (0-115 IU/ml)**	**TPOAb (0-9 IU/ml)**	**TSH (0.34-5.6 uIU/ml)**	**FT3 (2.5-3.9 pg/ml)**	**FT4 (0.58-1.46ng /dl)**	**T3 (0.87-1.78 ng/ml)**	**T4 (6.09-12.23 ug/dl)**
HT12	F	43	Thyroid nodule &HT	**401**	**97.4**	1.62	2.95	0.73	0.89	6.96
HT13	F	61	PTC&HT	**610.4**	**46.4**	1.95	3.62	0.77	12.9	8.53
HT14	F	60	PTC &HT	**875.2**	6.7	4.94	3.89	0.92	0.96	7.2
HT28	F	60	Thyroid nodule &HT	**>4000**	7.5	**6.05**	3.27	0.9	1.06	9.36
HT29	F	31	PTC &HT	**1229**	0.6	1.28	3.2	0.96	0.99	8.22

F female, *PTC* papillary thyroid carcinoma, *HT* Hashimoto's thyroiditis, *TGAb* thyroglobulin antibodies, *TPOAb* thyroperoxidase antibodies, *TSH* thyroid stimulating hormone, *FT3* free triiodothyronine, *FT4* free thyroxine, *T3* triiodothyronine, *T4* thyroxine

Supplementary Table2. Antibody information

Antibody	Company	Catalog number	Dilution	Host	website
VWF	CST	65707T	1：500	rabbit	https://www.cst-c.com.cn/products/primary-antibodies/vwf-d8l8g-xp-rabbit-mab/65707?site-search-
CD45	CST	13917T	1：200	rabbit	https://www.cst-c.com.cn/products/primary-antibodies/cd45-d9m8i-xp-rabbit-mab/13917?site-search-type=Products&N=4294956287&Ntt=13917s&fromPage=plp&_requestid=196936
ACTA2	CST	56856S	1：250	mouse	https://www.cst-c.com.cn/products/primary-antibodies/a-smooth-muscle-actin-1a4-mouse-mab-ihc-formulated/56856?site-search-type=Products&N=4294956287&Ntt=56856s&fromPage=plp&_requestid=197008
COL1A1	CST	72026S	1:100	rabbit	https://www.cellsignal.cn/products/primary-antibodies/col1a1-e8f4l-xp-rabbit-mab/72026?site-search-type=Products&N=4294956287&Ntt=72026s&fromPage=plp&_requestid=238421
ACKR1	Novus	NBP1-77278	1:400	rabbit	https://www.novusbio.com/products/darc-antibody_nbp1-77278
CD36 p	Abclonal	A1470	1:100	rabbit	https://abclonal.com.cn/catalog/A1470
CD3E	Abclonal	A1753	1:50	rabbit	https://abclonal.com.cn/catalog/A1753
FSP1	CST	13018	1:800	rabbit	https://www.cellsignal.cn/products/primary-antibodies/s100a4-d9f9d-rabbit-mab/13018?site-search-type=Products&N=4294956287&Ntt=13018&fromPage=plp&_requestid=238130
MECAC-79	Santa Cruz	sc-19602	1:50	Rat	https://www.scbt.com/p/meca-79-antibody-meca-79
CD20	Abclonal	A4893	1:100	rabbit	https://abclonal.com.cn/catalog/A4893
