# Supplementary material for: Lymphocyte infiltration and thyrocyte destruction are driven by stromal and immune cell components in Hashimoto’s thyroiditis
Source: Nat Commun. 2022 Feb 9;13:775. doi: 10.1038/s41467-022-28120-2 (PMC8828859; doi:10.1038/s41467-022-28120-2)
Supplement: Supplementary file 3 — Description of Additional Supplementary Files [file 41467_2022_28120_MOESM3_ESM.pdf]

Description of Additional Supplementary Files

Supplementary Data 1. Marker genes of cell clusters and subclusters of the thyroid

Supplementary Data 2. Marker genes of subclusters of stromal cells in thyroid tissues of HT patients

Supplementary Data 3. P values and means in CellphoneDB analysis results

Supplementary Data 4. Results of differential expression gene analysis between HT and non-HT patients by Bulk-RNAseq and GSVA score analysis

Supplementary Data 5. Marker genes of T cell subclusters in the merged immune cell data from thyroid tissues and PMBCs

Supplementary Data 6. Marker genes of B cell subclusters in the merged immune cell data from thyroid tissues and PMBCs

Supplementary Data 7. Marker genes of myeloid cell subclusters in the merged immune cell data from thyroid tissues and PMBCs

Supplementary Data 8. Differentially expressed genes and p values of M4 vs. M7 and M5 vs. M6
