# Supplementary material for: Lymphocyte infiltration and thyrocyte destruction are driven by stromal and immune cell components in Hashimoto’s thyroiditis
Source: Nat Commun. 2022 Feb 9;13:775. doi: 10.1038/s41467-022-28120-2 (PMC8828859; doi:10.1038/s41467-022-28120-2)
Supplement: Supplementary file 12 — Reporting Summary [file 41467_2022_28120_MOESM12_ESM.pdf]

nature research

Corresponding author(s): Huai-Dong Song
Last updated by author(s): Mar 1, 2021

Reporting Summary

Nature Research wishes to improve the reproducibility of the work that we publish. This form provides structure for consistency and transparency in reporting. For further information on Nature Research policies, see our Editorial Policies and the Editorial Policy Checklist.

Statistics

For all statistical analyses, confirm that the following items are present in the figure legend, table legend, main text, or Methods section.

n/a	Confirmed	
☐	☒	The exact sample size (*n*) for each experimental group/condition, given as a discrete number and unit of measurement
☐	☒	A statement on whether measurements were taken from distinct samples or whether the same sample was measured repeatedly
☐	☒	The statistical test(s) used AND whether they are one- or two-sided<br>*Only common tests should be described solely by name; describe more complex techniques in the Methods section.*
☐	☒	A description of all covariates tested
☐	☒	A description of any assumptions or corrections, such as tests of normality and adjustment for multiple comparisons
☐	☒	A full description of the statistical parameters including central tendency (e.g. means) or other basic estimates (e.g. regression coefficient) AND variation (e.g. standard deviation) or associated estimates of uncertainty (e.g. confidence intervals)
☐	☒	For null hypothesis testing, the test statistic (e.g. *F*, *t*, *r*) with confidence intervals, effect sizes, degrees of freedom and *P* value noted<br>*Give P values as exact values whenever suitable.*
☒	☐	For Bayesian analysis, information on the choice of priors and Markov chain Monte Carlo settings
☒	☐	For hierarchical and complex designs, identification of the appropriate level for tests and full reporting of outcomes
☒	☐	Estimates of effect sizes (e.g. Cohen's *d*, Pearson's *r*), indicating how they were calculated

Our web collection on statistics for biologists contains articles on many of the points above.

Software and code

Policy information about availability of computer code

Data collection	Image data was collected using NIS Elements (Nicon, V4.50.00)
Data analysis	All the codes are available under accession code 4575509 (https://zenodo.org/).<br>SPSS 22.0 software was used for student's t test. Originpro 8.5.1 was used for histograms plotting. Imaris 9.0.1 was used for analysis of image data. For single cell RNA sequencing, data was processed using the 10x Genomic Cell Ranger (V3.0.1). Batch effects were removed by harmony package (V1.0). Further analysis was conducted in R (V3.6.1) using Seurat (V3.1.1). Marker gene scores were determined using Monocle3 (V0.2.0). The marker significance test was conducted using R package lme4 (v1.1.27) and lmerTest(v3.1.3). GSVA analysis was performed by GSVA package (V1.34.0). Cell–cell interactions analysis was performed by CellphoneDB (V2.0). Plots were generated using ggplot2 packages (V3.2.1) in R.

For manuscripts utilizing custom algorithms or software that are central to the research but not yet described in published literature, software must be made available to editors and reviewers. We strongly encourage code deposition in a community repository (e.g. GitHub). See the Nature Research guidelines for submitting code & software for further information.

Data

Policy information about availability of data

All manuscripts must include a data availability statement. This statement should provide the following information, where applicable:

- Accession codes, unique identifiers, or web links for publicly available datasets
- A list of figures that have associated raw data
- A description of any restrictions on data availability

Single-cell sequencing data and bulk RNA sequencing data generated in this study have been deposited in the CNGB database under accession code CNP0001494

(https://db.cngb.org/). Known receptor-ligand interactions can be downloaded from cellphoneDB (http://www.cellphonedb.org/). Human reference (GRCh38) dataset required for Cell Ranger can be downloaded at 10x Genomics (https://cf.10xgenomics.com/supp/cell-exp/refdata-gex-GRCh38-2020-A.tar.gz). All other data generated in this study are presented in the Supplementary data or source data provided with this paper.

Field-specific reporting

Please select the one below that is the best fit for your research. If you are not sure, read the appropriate sections before making your selection.

☒ Life sciences ☐ Behavioural & social sciences ☐ Ecological, evolutionary & environmental sciences

For a reference copy of the document with all sections, see nature.com/documents/nr-reporting-summary-flat.pdf

Life sciences study design

All studies must disclose on these points even when the disclosure is negative.

Sample size	No statistical method was used to predetermine sample size. Sample sizes were chosen based on sample availability and fundation
Data exclusions	the single-cell RNA sequencing data of the thyroid tissue from patient HT14 was excluded from the study because the extremly low capture rate of cells.
Replication	Cell experiments were performed for at least three times with consistent results. HE staining, polychromatic immunofluorescence staining and RNAscope detections were confirmed in at least three biological replicates with consistent results.
Randomization	HT and non-HT groups were divided according to well-defined diagnosis criteria as provided in the method section. We only compare the expression profile between the thyroid of HT and non-HT groups and no medication or treatment was given in patients to observe the effect. Thus, No method of randomization was used.
Blinding	The doctors for pathological diagnosis were blinded to patient information during section assessment. For other experiments, blinding was not relevant because the results generated in these experiments are either analyzed quantitatively, or analyzed according to standard procedures or presented directly and are hardly influenced by performers' will.

Reporting for specific materials, systems and methods

We require information from authors about some types of materials, experimental systems and methods used in many studies. Here, indicate whether each material, system or method listed is relevant to your study. If you are not sure if a list item applies to your research, read the appropriate section before selecting a response.

Materials & experimental systems

n/a	Involved in the study	
☐	☒	Antibodies
☐	☒	Eukaryotic cell lines
☒	☐	Palaeontology and archaeology
☒	☐	Animals and other organisms
☐	☒	Human research participants
☒	☐	Clinical data
☒	☐	Dual use research of concern

Methods

n/a	Involved in the study	
☒	☐	ChIP-seq
☒	☐	Flow cytometry
☒	☐	MRI-based neuroimaging

Antibodies

Antibodies used	VWF (CST, 65707T, 1:500, rabbit); CD45 (CST , 13917T, 1:200, rabbit); ACTA2(CST, 56856S, 1:250, mouse); COL1A1 (CST, 72026S, 1:100, rabbit); ACKR1(Novus, NBP1-77278, 1:400, rabbit); CD36 (Abclonal, A1470, 1:100, rabbit); CD3E(Abclonal, A1753, 1:50, rabbit), FSP1 (CST, 13018, 1:800, rabbit); MECAC-79 (Santa Cruz, sc-19602, 1:50, rat); CD20 (Abclonal, A4893, 1:100, rabbit)
Validation	Antibodies were validated for IHC in Human tissues as noted on manufacturer's website as cited in supplementary table 2 and as listed below. Additional validation was done by the use of negative control samples in each experiment. VWF(https://www.cst-c.com.cn/products/primary-antibodies/vwf-d8l8g-xp-rabbit-mab/65707?site-search-)<br>CD45(https://www.cst-c.com.cn/products/primary-antibodies/cd45-d9m8i-xp-rabbit-mab/13917?site-search-type=Products&N=4294956287&Ntt=13917s&fromPage=plp&_requestid=196936)<br>ACTA2 (https://www.cst-c.com.cn/products/primary-antibodies/a-smooth-muscle-actin-1a4-mouse-mab-ihc-formulated/56856?site-search-type=Products&N=4294956287&Ntt=56856s&fromPage=plp&_requestid=197008)<br>COL1A1(https://www.cellsignal.cn/products/primary-antibodies/col1a1-e8f4l-xp-rabbit-mab/72026?site-search-type=Products&N=4294956287&Ntt=72026s&fromPage=plp&_requestid=238421)<br>ACKR1 (https://www.novusbio.com/products/darc-antibody_nbp1-77278)<br>CD36(https://abclonal.com.cn/catalog/A1470)

CD3E(https://abclonal.com.cn/catalog/A1753)
FSP1(https://www.cellsignal.cn/products/primary-antibodies/s100a4-d9f9d-rabbit-mab/13018?site-search-type=Products&N=4294956287&Ntt=13018&fromPage=plp&_requestid=238130)
MECAC-79(https://www.scbt.com/p/meca-79-antibody-meca-79)
CD20(https://abclonal.com.cn/catalog/A4893)

Eukaryotic cell lines

Policy information about cell lines

Cell line source(s)	C2C12 mouse myoblasts were obtained from ATCC, and T8.1 were obtained from the Chinese Academy of Science in Shanghai. Human umbilical vein endothelial cells (HUVECs) were obtained from FuHeng Cell Center
Authentication	The cell lines were not authenticated.
Mycoplasma contamination	All cell-lines were tested negative for mycoplasma.
Commonly misidentified lines (See ICLAC register)	None of the cell line is listed in ICLAC Register of Misidentified Cell Lines.

Human research participants

Policy information about studies involving human research participants

Population characteristics	HT patients for single cell RNA sequencing were all females, the age of HT12, HT13, HT14, HT28 and HT29 is 43, 61, 60, 60, and 31 respectively (supplementary table1). Characteristics of 16 HT and 50 non-HT patients for bulk-RNAseq are provided in supplementary data 4. Among these 66 patients, 47 were female and 19 were male, and the average age is 48.5.
Recruitment	Patients were all recruited from the Shanghai Ninth People's Hospital. Five HT patients with thyroid neoplasms requiring thyroidectomy with elevated TGAb or TPOAb were included for scRNA-seq. Considering the bias may be caused by small patient number, important findings were further confirmed in by conducting bulk RNA-seq of the thyroid tissues in sixteen HT patients and 50 non-HT patients as well as by staining experiments in more patients.
Ethics oversight	The study was performed in accordance with protocols approved by the ethics committee of Shanghai Ninth People's Hospital

Note that full information on the approval of the study protocol must also be provided in the manuscript.
